# Current positioning and future development of belantamab mafodotin and other antibody-drug conjugates to treat multiple myeloma

**DOI:** 10.3389/fonc.2026.1822419

**Published:** 2026-06-10

**Authors:** Giuseppe Bertuglia, Alessandro Di Nicola, Francesca Gay, Alessandra Larocca, Benedetto Bruno, Mattia D’Agostino

**Affiliations:** Division of Hematology, Azienda Ospedaliera Universitaria (AOU) Città della Salute e della Scienza di Torino, Department of Molecular Biotechnology and Health Sciences, University of Torino, Turin, Italy

**Keywords:** antibody-drug conjugate (ADC), B-cell maturation antigen (BCMA), belantamab mafodotin (Blenrep), multiple myeloma, treatment sequencing

## Abstract

The treatment of Multiple myeloma (MM) has been revolutionized by the advent of immunotherapies. Monoclonal antibodies targeting specific markers greatly contributed to patients’ outcome improvement. Antibodies can be conjugated with a toxic payload to exert anti-tumor efficacy (antibody–drug conjugates, ADCs). ADCs combine direct cytotoxicity with immune-mediated effects, potentially sustaining antimyeloma activity even with prolonged dosing intervals. B-cell maturation antigen (BCMA) is selectively expressed on plasma cells, including malignant clones and represents a validated target in MM. Belantamab-mafodotin is the first in class anti-BCMA ADC approved in MM. The drug demonstrated single agent activity in heavily pretreated MM patients enrolled in the phase II DREAMM-2 trial. However, the confirmatory phase III DREAMM-3 trial failed to show superiority over standard therapy, leading to a temporary withdrawal of its approval by regulatory agencies. Despite this setback, recent data from the DREAMM-7 and DREAMM-8 trials have renewed interest in Belantamab-mafodotin, showing a benefit over standard of care treatments when evaluated in earlier lines of therapy and in combination with known therapeutic backbones. Recently, other ADCs against BCMA or other targets are being developed. This review aims to explore the current clinical positioning of ADCs within MM therapeutic landscape and their possible future development.

## 1 Introduction

Antibody–drug conjugates (ADCs) represent an emerging therapeutic class in multiple myeloma (MM), designed to exploit tumor-associated antigens to deliver potent cytotoxic agents directly to malignant plasma cells (PCs). These agents consist of a monoclonal antibody (MoAb) directed against a selected surface antigen, a chemical linker, and a cytotoxic payload. The rationale underlying ADCs relies on the presence of target antigens that are highly expressed on clonal plasma cells but largely absent from normal tissues, enabling selective tumor-cell killing while limiting off-target toxicity. Among these antigens, B-cell maturation antigen (BCMA), a transmembrane receptor, has been the most extensively validated, given its strong and relatively restricted expression on late-stage B cells and plasma cells, and its central role in plasma-cell survival (1). Of note, many immunotherapeutic approaches towards BCMA have been developed so far (2, 3).

ADCs were among the first targeted immunotherapies to enter clinical testing in the relapsed/refractory setting and this class, with other immunotherapeutic classes like bispecific antibodies (bsAbs) and chimeric antigen receptor T-cell (CAR-T), are rapidly reshaping MM treatment paradigm and are the primary focus of contemporary clinical development. Compared to other immunotherapies, ADCs may be particularly interesting in patients who may be unsuitable for cellular therapies or bsAbs.

## 2 The first ADC in multiple myeloma: belantamab mafodotin

Belantamab mafodotin is an ADC, composed of a humanized IgG1 monoclonal antibody targeting BCMA, conjugated to the cytotoxic anti-microtubule agent monomethyl auristatin F. Upon binding to BCMA, the payload is internalized by the plasma cell, leading to intracellular release of the cytotoxin and subsequent cell death (4). However, this is not its only mechanism of action. The fragment crystallizable (Fc) region of the antibody can also engage Fc receptors on macrophages, thereby triggering immune-mediated mechanisms such as antibody-dependent cellular cytotoxicity (ADCC), complement-dependent cytotoxicity (CDC), and phagocytosis. Interestingly, Fc receptors are also expressed on myeloma cells themselves, potentially offering an additional route for drug entry even in cases previously exposed to other BCMA-directed therapies (4).

### 2.1 The troubled journey of approval

Belantamab mafodotin was introduced during a critical juncture in the treatment landscape of MM, particularly for patients who were triple- or penta-refractory, including immunomodulatory drugs (IMiDs), proteasome inhibitors (PIs), and anti-CD38 MoAbs. At the time, therapeutic options for this population were extremely limited, and the prognosis was dismal, with median overall survival (OS) often measured in months (5, 6). In this context of urgent unmet clinical need, belantamab mafodotin offered a novel mechanism of action and a much-needed sense of therapeutic renewal.

The phase II DREAMM-2 trial provided encouraging efficacy data, demonstrating clinically meaningful response rates and manageable toxicity in heavily pretreated patients (7). The trial was designed to randomize patients to receive 2.5 mg/kg or 3.4 mg/kg of belantamab mafodotin via intravenous infusion every 3 weeks until disease progression or unacceptable toxicity. The median overall response rate (ORR) was similar between the two dose groups (31% vs 34%), just like PFS (2.9 months vs 4.9 months) and ocular toxicity (27% vs 21%), while the grade 3-4 (G3-4) infection rate was significantly higher in the 3.4 mg/kg cohort (11% vs 4% in the 2.5 mg/kg cohort). OS and median duration of response (DoR) were 11 and 13.7 months in the 2.5 mg/kg cohort, while not reached in the 3.4 mg/kg cohort. Comparing the risk-benefit profile of both dosing schedules, the DREAMM-2 trial favored the 2.5 mg/Kg dosage. These results supported the Food and Drug Administration’s (FDA) decision to grant accelerated approval in 2020 for patients who have received at least 4 prior therapies, including an anti-CD38, a PI, and an IMid (8). The European Medicines Agency (EMA) subsequently granted conditional approvals. Belantamab mafodotin thus became an important treatment option in relapsed/refractory multiple myeloma (RRMM), particularly for patients with no remaining standard-of-care alternatives (9).

However, the confirmatory phase III DREAMM-3 trial, which compared belantamab mafodotin single agent (SA) to pomalidomide plus dexamethasone (Pd) in patients with RRMM (10), failed to demonstrate a statistically significant improvement in PFS, raising concerns about its efficacy when benchmarked against existing therapies. Consequently, the FDA withdrew its approval in 2022, citing the failure to confirm clinical benefit in the phase III setting. This decision was soon followed by regulatory authorities in Europe (EMA), leading to the drug’s removal from the market.

The belantamab mafodotin experience reflects the dynamic and sometimes fragile trajectory of drug development in oncology, particularly when accelerated approval mechanisms are employed. Nevertheless, belantamab mafodotin’s early success paved the way for further development of BCMA-targeting strategies and for exploring their role in earlier therapeutic lines and in combination with known anti-MM backbones.

The phase III DREAMM-7 (11) and DREAMM-8 (12) trials have provided new clinical evidence supporting belantamab mafodotin use in combination with bortezomib and dexamethasone (BelaVd) and with pomalidomide and dexamethasone (BelaPd), respectively. These combinations have shown improved efficacy compared to standard regimens in patients who have received one or more prior lines of therapy. The median PFS was 36.6 months for BelaVd, significantly higher than Daratumumab-Vd (DaraVd) control arm in the DREAMM-7 trial (11). In DREAMM-8, BelaPd produced a median PFS of 32.6 months that was significantly superior to Pomalidomide-Vd (PVd) control arm (12) (*details in* **Table 1**). In DREAMM-7, belantamab was initially administered at 2.5 mg/kg every 3 weeks, in line with DREAMM-2, with dose reductions to 1.9 mg/kg or interval extensions based on toxicity (11). In DREAMM-8, only the first cycle used 2.5 mg/kg; subsequent cycles were fixed at 1.9 mg/kg every 4 weeks, delayable every 8 weeks in case of toxicities (12); both trials demonstrated a decrease of ocular events with dose reduction and/or dose frequency reduction, reverting the adversities in most cases.

**Table 1:** Efficacy and safety profile in relapsed/refractory multiple myeloma or newly diagnosed multiple myeloma treated with belantamab mafodotin (monotherapy or combinations) in the main clinical trials and real-world settings.

Trial	Bela dosing schedule	mORR (%)	mDoR (months)	mPFS (months)	mOS (months)	Dara exposed/refractory (%)*	Len exposed/refractory (%)*	BCMA refractory (%)*	Toxicity G3-4 (%)
RRMM									
DREAMM-2 (7) Bela SA	2.5 vs 3.4 mg/kg iv q3w	31 vs 34	11 vs NR	2.9 vs 4.9	13.7 vs NR	100/100**	100/90	0	Ocular 27 vs 21 Infections 4 vs 11
DREAMM-3 (10) Bela SA vs Pd	2.5 mg/kg iv q3w	41 vs 36	NR	11.2 vs 7	21.2 vs 21.1	40/33	100/71	0	Ocular 29 Infections 13 vs 25
DREAMM-7 (11) BelaVd vs DVd	2.5 mg/kg iv q3w	83 vs 71	35.6 vs 17.8	36.6 vs 13.4	NR vs NR (HR 0.55) [median follow-up 39 months]	1/0	52/34	0	Ocular 34 Infections 31 vs 20
DREAMM-8 (12) BelaPd vs DPd	2.5 mg/kg iv q4w (Cycle 1); 1.9 mg/kg iv q4w (Cycle 2 onward)	77 vs 72	NR vs 17.5	32.6 vs 12.5	NR vs NR (HR 0.77) [median follow-up 22 months]	23/21	100/81	0	Ocular 43 Infections 49 vs 26
Real world Bela SA (Talbot et al) (26)	2.5 mg/kg iv q3w	38.1	9	3.5	9.3	100/61.9	100/44.3	0	Ocular 40.8 Infections NA
Real world Bela SA (Mewalalla et al) (27)	2.5 mg/kg iv q3w	40	13	5	12	NA	NA	15	Ocular 43 Infections 17
Real world Bela SA (Shragai et al) (28)	2.5 to 3.4 mg/kg iv wq3	45.5	8.1	4.7	14.5	95.2	91.5	0	Ocular 40 Infections 7.5
NDMM									
DREAMM-9 BelaVRd (15)	1.9 mg/kg iv q3/4w vs 1.9 mg/kg iv q6/8w vs 1.4 mg/kg iv q6/8w	100	NR	NR	NR	Not applicable	Not applicable	Not applicable	Ocular 83 vs 92 vs 75 Infections 42 vs 58 vs 25
NCT04808037 BelaRd trial (16)	2.5 mg/kg iv q8w vs 1.9 mg/kg iv q8w vs 1.4 mg/kg iv q8w vs	97.6	NR	18-month PFS 83%	NR	Not applicable	Not applicable	Not applicable	Ocular 75 vs 58.3 vs 33.3 Infections overall 30%
GEM-BelaVRd (20)	2.5 mg/kg iv q8w during induction and consolidation; 1.9 mg/kg ev q8w during maintenance	96	NA	36-month PFS 78%	36-month OS 82%	Not applicable	Not applicable	Not applicable	Ocular 51.2 Infections 26

Safety is expressed by the most important-related adverse events: ocular toxicity and infection. *percentages about daratumumab, anti-BCMA and lenalidomide exposition and refractoryness refers only to groups treated with belantamab in every study, and not to the control groups. **Percentes refers to the dosage 2.5 mg/Kg, which is the approved one.

RRMM, relapsed-refractory multiple myeloma; NDMM, newly diagnosed multiple myeloma; IV, intravenous; q(number)w, every (number) weeks; m, median; Bela, belantamab; SA, single agent; BelaVd, belantamab-velcade-dexamethasone; BelaPd, belantamab-pomalidomide-dexhametasone; DVd, daratumumab-velcade-dexamethasone; DPd, daratumumab-pomalidomide-dexamethasone; BelaRd, belantamab-lenalidomide-dexamethasone; BelaVRd, belantama-velcade-lenalidomide-dexamethasone; ORR, overall response rate; DoR, duration of response; PFS, progression free survival; OS, overall survival; Dara, daratumumab; Len, lenalidomide; BCMA, B-cell maturation antigen; NR, not reached; NA, not available; HR, hazard ratio; G3-4, grade 3 or 4.

These results, combined with dose adjustments, were pivotal for the approval by EMA, for both the combinations in RRMM from second line of therapy, in July 2025. On October 23, 2025, the FDA approved Bela-Vd for RRMM who have received at least two prior lines of therapy, including PIs and IMiDs (13). As a result, belantamab mafodotin combinations are now included in the treatment algorithm for RRMM in the second line with a level “1” of evidence and with an “A” grade of recommendation (14).

Regarding first line treatment, data on small early phase clinical trials are available. The DREAMM-9 trial (15) and the NCT04808037 clinical trial (16) are two randomized, phase 1/2 studies, evaluating multiple doses and schedules of belantamab mafodotin in combination with VRd (BelaVRd) and Rd (BelaRd), respectively, in patients with non–transplant-eligible (NTE) newly diagnosed (ND) MM. High overall response rates (near to 100% in both the studies) were observed across all cohorts, and rates of treatment discontinuations due to grade 3/4 ocular events were low, indicating these events could be managed with dose modifications (*details’ summary in* **Table 1**). Moreover, in a dose expansion phase of the NCT04808037 clinical trial, two safety monitoring strategies were compared: ophthalmologist-assessed ocular adverse events vs hematologist-lead vision-related anamnestic tool. Interestingly, grade ≥2 ocular adverse events were no different among the two approaches, paving the way for a possible hematologist-centered monitoring of belantamab mafodotin adverse events (16).

These favorable results led to the design of DREAMM-10 phase 3 trial, randomizing NTE NDMM patients to BelaRd vs DaraRd upfront (17). If positive, this study has the potential to bring belantamab mafodotin frontline. However, it is important to note that the trial comparator (DaraRd) may soon be outperformed by emerging quadruplet regimens, such as isatuximab–bortezomib–lenalidomide–dexamethasone (IsaVRd) and daratumumab-bortezomib-lenalidomide-dexamethasone (DaraVRd), in the NTE NDMM setting (18, 19). This evolving treatment landscape could potentially limit its approval in first line setting by regulatory agencies.

The GEM-BelaVRd (20) phase 2 trial is currently expanding the role of BelaVRd as induction for transplant-eligible (TE) NDMM: belantamab mafodotin was administered every 8 weeks for 6 cycles, then patients underwent autologous stem cells transplantation followed by 2 Bela-VRd consolidation cycles and a Bela-R maintenance until progression (*details in* **Table 1**). After a median follow-up of 3 years, the percentage of CR or better was higher than 80%, with a favorable safety profile, where eye-related side effects were common, but reversible and manageable with dose modifications/interruptions of belamaf (20).

### 2.2 Promises and expectations of ADCs combinations in RRMM

Regardless of the results of DREAMM-7 and DREAMM-8, the therapeutic landscape has evolved considerably since belantamab mafodotin’s initial introduction, largely due to the emergence and regulatory approval of new combinations upfront. Thus, it is important to consider the patient populations enrolled in the DREAMM-7 (11) and DREAMM-8 (12) trials.

Currently, most NTE patients relapse after first-line therapy with DaraRd (21), making them refractory to both anti-CD38 and lenalidomide. Following the approval of DaraVRd and IsaVRd in the NTE first line setting (14), second line RRMM patients may be proteasome inhibitor–exposed or refractory as well. TE patients typically relapse after induction with anti-CD38-based quadruplets followed by lenalidomide +/- Daratumumab maintenance (22–24). As a consequence, at relapse they are usually bortezomib-exposed, daratumumab exposed or refractory and lenalidomide-refractory at first progression. In DREAMM-7, only about half of patients had prior exposure to lenalidomide (35% were refractory), while daratumumab-refractory patients were excluded (11). A subanalysis of DREAMM-7 focusing on second-line lenalidomide-refractory patients still showed a benefit of BelaVd vs DaraVd, with a median PFS of 35.7 months in Bela-Vd treated patients. However, more data are needed to understand if data in this trial will translate into real-world effectiveness, particularly in daratumumab and lenalidomide double refractory patients (25). Notably, only ~10% of patients in DREAMM-7 had prior exposure to carfilzomib or pomalidomide, making it difficult to generalize these results to later-line.

In contrast, in DREAMM-8, all patients had prior lenalidomide exposure (81% were refractory), but only ~20% were exposed or refractory to daratumumab (12). Thus, even though DREAMM-8 may more closely reflect the current treatment landscape, it still does not fully capture the complexity of later-line, triple-class-refractory patients, who represented only ~25% of the study population. Moreover, both trials excluded patients who had prior treatment with other anti-BCMA agents, a sequencing that is increasingly common in clinical practice.

Real-world data, with a special focus on daratumumab- and lenalidomide-refractory patients at first relapse will be essential to confirm the efficacy observed in clinical trials and to better define their role in the actual therapeutic landscape. At present, the only available real-world evidence concerns belantamab mafodotin monotherapy. Two retrospective real-world studies, in which most patients would not have met the eligibility criteria of the DREAMM-2 and DREAMM-3 trials and were characterized by high-risk disease features, reported efficacy outcomes broadly consistent with those observed in DREAMM-2. Specifically, median PFS ranged between 3.5 and 5 months (compared with 2.9 months in DREAMM-2), while median OS was 9.3 and 12 months, respectively (13.7 months in DREAMM-2) (7, 26, 27). Across both real-world studies and clinical trials, the presence of extramedullary disease (EMD) and BCMA-refractory disease was consistently associated with inferior outcomes, whereas deeper responses were associated with improved survival outcomes. In DREAMM-3 (10), the population was less heavily pretreated with a median number of prior lines in Belantamab mafodotin SA arm of 4, 40% had been exposed to anti-CD38 therapy (vs a median number of prior lines of 7, with 100% of patients anti-CD38 exposed and refractory in DREAMM-2). Another real-world study including patients with a median of 4 prior lines of therapy reported a median OS of 14.5 months (28) (vs 21.2 in DREAMM-3 (10)). These real-world studies, consistently with the findings from DREAMM-2 and DREAMM-3, suggest that belantamab mafodotin may induce favorable outcomes when used earlier in the disease course, in less heavily pre-treated populations (29).

**Table 1** summarizes the main results of clinical trials and real-world studies.

### 2.3 The safety profile

Evaluating advantages and disadvantages of each therapy, in accordance with official guidelines, is essential in selecting the most appropriate anti-BCMA agent for the patient. Belantamab mafodotin is associated with a well-recognized, class-specific ocular toxicity, most commonly manifesting as keratopathy, blurred vision, or dry eye. Some ADCs may reach the cornea either through the tear film or via the vascularized limbal region and can induce ocular toxicity through both on-target and off-target mechanisms: on-target toxicity occurs when non-malignant cells expressing the target antigen bind the monoclonal antibody, leading to intracellular release of the cytotoxic payload; off-target toxicity, conversely, results from premature deconjugation of the payload mediated by circulating enzymes, with subsequent uptake by neighboring cells through endocytosis or macropinocytosis. In the case of belantamab mafodotin, ocular toxicity is thought to be primarily driven by macropinocytosis of the ADC by corneal epithelial cells, including limbal epithelial stem cells. Internalization of the cytotoxic payload leads to epithelial cell damage, and subsequent centripetal migration of injured cells contributes to keratopathy and visual symptoms. Histopathological analyses in patients with RRMM have demonstrated corneal epithelial cells containing intracytoplasmic inclusions, apoptosis, and occasional inflammatory changes following belantamab mafodotin exposure. The detection of ADCs in tear fluid and blood, together with the pattern of corneal involvement, supports the limbal vasculature as a key route of corneal exposure (30).

In DREAMM-2 and DREAMM-3 trials, belantamab mafodotin SA showed any grade ocular toxicity rate ~ 70%, mainly due to blurred vision. Of note, visual changes were reversible and manageable in the majority of cases (10). Ocular toxicity incidence and grading was comparable in real-world experiences (26–28), which means in real life the management of ophthalmological side effects seem reversible and manageable. In the DREAMM-7 and DREAMM-8 trials, patients treated with belantamab mafodotin underwent regular ophthalmic examinations, with ocular examination findings (OEFs) graded according to the KVA scale. Per protocol, grade ≥2 OEFs required temporary treatment interruption until resolution. The incidence of grade ≥2 OEFs was high, occurring in 86% of patients receiving BelaVd and 87% of those treated with BelaPd. Importantly, dose modifications allow effective management of ocular toxicity while maintaining clinical benefit, typically through reduced dosing frequency or prolonged intervals between belantamab mafodotin infusions (every 8–12 weeks) (11, 12). Ophthalmologic evaluation is recommended before each infusion during the first four cycles, and the treating physician may adjust or delay subsequent doses based on vision-related assessments (14). These strategies, including dose delays and reductions, facilitate recovery from ocular adverse events without compromising the drug’s efficacy. Indeed, in first-line trials, Belantamab dosage (1.9 and 1.4 mg/kg) and schedule (every 8 weeks) is modified to reduce the incidence of ocular toxicity (17, 20).

Although ocular toxicity represents the most recognized adverse event associated with belantamab mafodotin, emerging reports suggest that proteinuria should also be considered a potential drug-related toxicity. In the reported cases, proteinuria was predominantly selective and of glomerular origin, mainly characterized by albuminuria (31). Although the underlying mechanism remains unclear, it can be hypothesized that the microtubule-disrupting activity of mafodotin may induce podocyte injury, resulting in increased glomerular permeability. This hypothesis is supported by preclinical data showing dose-dependent glomerular proteinuria and by evidence of BCMA expression on podocytes in certain inflammatory settings (32).

“On-target, off-tumor” toxicities common to anti-BCMA therapies include hypogammaglobulinemia and cytopenias, which translate into an increased risk of infections compared with other approved regimens. By targeting BCMA, a receptor expressed not only on malignant plasma cells but also on normal plasma cells and mature B cells, these treatments induce profound and often prolonged depletion of antibody-producing cells, resulting in impaired humoral immunity and susceptibility to infections.

However, the rate of grade 3–4 infections reported with SA belantamab mafodotin is approximately 10% (10), which is substantially lower than the incidence of severe and opportunistic infections observed with anti-BCMA CAR T-cell therapies (20–27% (33, 34)) or bsAbs (26–55% (35, 36)) in registrational trials. By contrast, in the DREAMM-7 and DREAMM-8 trials, where belantamab was administered in combination with other agents, the incidence of grade ≥3 infections was approximately 30% with Bela-Vd (11) and to 50% with Bela-Pd (12), indicating that the addition of concomitant therapies significantly increases susceptibility to infections.

### 2.4 The cost/effectiveness profile

Beyond clinical efficacy and safety, economic considerations and drug accessibility represent critical factors influencing the real-world implementation of belantamab mafodotin, particularly in low- and middle-income countries. As with many novel immunotherapies, the cost of ADCs remains substantial, driven by complex manufacturing processes, biologics development, and the need for specialized monitoring. A budget impact analysis conducted in the United States suggested that the introduction of belantamab mafodotin single agent in heavily pretreated RRMM patients may be approximately cost-neutral at the population level, largely due to the relatively small number of eligible patients and the offset of costs associated with alternative therapies and disease complications (37). However, in resource-limited environments, access to belantamab mafodotin is further challenged by the need for regular ophthalmologic assessments, drug supply logistics, and reimbursement limitations. While pharmaceutical company access programs and differential pricing strategies may partially mitigate these barriers, disparities in availability remain substantial across geographic regions (38). Moreover, when compared with other BCMA-targeted therapies such as CAR T-cell products, belantamab mafodotin may offer some logistical advantages due to its off-the-shelf availability and outpatient administration; nevertheless, its cost and infrastructure requirements still limit widespread adoption in less affluent healthcare systems. It is important to precise that all of these analyses were conducted on belantamab mafodotin used as monotherapy with dosing schedules based on the DREAMM-2 trial (7), and supplementary data on the emerging combinations are required.

### 2.5 Finding the right place for belantamab-based regimens: mechanism of resistance to anti-BCMA and anti-BCMA sequencing strategies

CAR-T cell therapies such as idecabtagene vicleucel (ide-cel) (34) and ciltacabtagene autoleucel (cilta-cel) (33), as well as bsAbs including teclistamab (36), elranatamab (35) and linvoseltamab (39) are FDA approved BCMA-targeting therapies that can be used in RRMM. Nevertheless, the patient population to be treated with different immunotherapeutic drugs may be not superimposable.

The optimal sequencing in the era of BCMA-targeted therapies is nowadays a central topic of discussion. The registrational studies of approved BCMA‐directed immunotherapies excluded patients who previously received BCMA‐directed therapy. The use of Bela-Vd and Bela-Pd in earlier lines of therapy may affect the subsequent efficacy or feasibility of other BCMA-targeted approaches, such as CAR T-cell therapies and bsAbs, which are currently considered the most effective treatment options for patients with RRMM. On the other hand, prior anti-BCMA CAR-T and bsAbs may affect anti-BCMA ADCs efficacy as well. To better inform sequencing decisions, it is essential to understand the biological role of BCMA and examine the differing mechanisms of action among available BCMA-targeted agents.

Recent molecular and genomic studies have identified several common mechanisms of resistance to BCMA-targeted therapies. In general, BCMA loss after treatment is not a common event, with BCMA expression remaining positive in most patients (40). The biallelic deletion of the TNFRSF17 gene, which encodes the BCMA antigen results in the complete loss of the therapeutic target. Additionally, point mutations in the TNFRSF17 gene may lead to epitope alterations that hinder effective drug binding (41). Furthermore, tumor cells may downregulate BCMA expression on their surface or undergo proteolytic cleavage of BCMA by gamma-secretase, resulting in reduced surface availability and increased levels of soluble BCMA (sBCMA). This soluble form of BCMA may act as a decoy receptor, a phenomenon especially prevalent in patients with high tumor burden (42). In this context, pharmacological strategies aimed at increasing BCMA surface expression are under active investigation. In particular, inhibition of gamma-secretase has been shown to reduce BCMA shedding and increase membrane-bound BCMA density on plasma cells, thereby potentially enhancing the efficacy of BCMA-targeted therapies, including ADCs. Early-phase clinical studies combining gamma-secretase inhibitors with anti-BCMA agents have demonstrated promising biological activity, supporting this approach as a potential strategy to overcome resistance and improve treatment responses (43, 44). Notably, elevated sBCMA levels are more commonly associated with primary resistance or early relapse, rather than with long-term treatment failure (45). Other resistance mechanisms include the exhaustion or senescence of T-cells, which particularly compromises the efficacy of CAR-T and bsAbs therapies. In this context, the endogenous immune microenvironment may interact with CAR T-cell therapy, influencing treatment durability and potentially contributing to either sustained responses or early resistance (46). Similarly, a treatment-free interval during bispecific antibody therapy may ameliorate or prevent T-cell exhaustion (47, 48).

Not all mechanisms carry the same clinical impact. For example, biallelic deletion of TNFRSF17 confers permanent resistance, but remains relatively rare. In contrast, downregulation or cleavage of BCMA are more frequent and may be transient. A sufficiently long washout period may allow BCMA re-expression on the cell surface, potentially restoring sensitivity to subsequent targeted therapies (49). Therefore, proven resistance to a BCMA-targeted therapy does not necessarily preclude a response to subsequent agents targeting the same antigen. This concept is well established in multiple myeloma, as patients previously treated with bortezomib may still respond to carfilzomib (50) or those exposed to lenalidomide benefits from pomalidomide despite the shared targets (51). However, this appears to be less consistently true for anti-CD38 antibodies such as daratumumab and isatuximab, where, despite binding to different epitopes, the clinical efficacy of sequencing remains limited (52). Combining belantamab mafodotin with standard anti-myeloma backbones as shown in GEM-BelaVRd or DREAMM-10 trials or novel agents (e.g., gamma-secretase inhibitors) may decrease the selective pressure on BCMA for efficacy and potentially extend the efficacy of subsequent BCMA-targeted therapies (17, 20, 42).

In general, if treatment with a second BCMA-targeted therapy is considered, retained target expression should be confirmed by flow cytometry or immunohistochemistry (53). In addition, assessment of soluble BCMA (sBCMA) levels may be incorporated into clinical practice to help predict response to subsequent BCMA-directed treatment following prior exposure to the same target (54). Available evidence suggests that patients previously treated with belantamab mafodotin may experience inferior responses to subsequent anti-BCMA CAR T-cell therapies or bsAbs compared with BCMA-naïve patients, with a substantial reduction in treatment efficacy (55–57) (**Table 2**). The underlying mechanisms are not yet fully understood but may include antigen escape, reduced BCMA density, or epitope masking (41, 49). Importantly, several studies suggest that the introduction of a BCMA-free interval may partially mitigate the negative impact of prior BCMA-directed therapy on the efficacy of subsequent BCMA-targeting bsAbs; however, this observation has not been consistently confirmed (55, 56), and further studies are required. Regarding the use of belantamab mafodotin after other BCMA-directed therapies, limited data are available from retrospective studies with heavily pretreated MM patients who received single‐agent belantamab mafodotin; some studies show reduced activity of belantamab mafodotin after previous BCMA‐directed therapy, while others show similar activity of belantamab mafodotin in patients with or without previous BCMA exposure (49). BCMA-targeting BsAbs may impair T-cell fitness, negatively affecting CAR T-cell manufacturing through reduced expansion and lower transduction efficiency as well as the cytotoxic capacity of the resulting CAR T-cell products against MM cells (58). By contrast, this mechanism does not appear to compromise the activity of belantamab mafodotin, whose efficacy primarily depends on BCMA expression on tumor cells rather than on T-cell functionality. In addition, Belantamab mafodotin promotes the proliferation of CD4^+^ T cells and intratumoral regulatory T cells, significantly increases the infiltration of CD8^+^ and CD4^+^ T cells (59).

**Table 2:** Efficacy of anti-BCMA retreatment in relapsed/refractory multiple myeloma, underlining a confrontation between using belantamab-mafodotin after other anti-BCMA agents and viceversa.

Previous Bela exposed/general population (n°)	Subsequent anti-BCMA treatment	Outcomes (vs general population)
13/56	Cilta-cel (CARTITUDE-2 cohort C) (57)	ORR: 61.5% (vs > 95%); DoR: 11.5 (vs > 18) months
29/165	Teclistamab (Majestec-1 cohort C) (57)	ORR: 55.2% (vs 63%); DoR: 14.8 (vs 24)months; PFS: 7.3 (11.4) months; OS: 16 (vs 22.2) months
59/NA	Elranatamab (pooled data from MagnetisMM-1,-3,-9) (57)	ORR: 41.4%; DoR at 9 months 67.3% (general population not evaluable)
37/NA	Ide-cel (real world Ferreri et al) (57)	ORR 68% (general population not evaluable)
23	Any anti-BCMA (real world) (56)	14 CAR-T ☮ ORR 79%, PFS 7 months 9 Teclistamab ☮ ORR 44%, PFS 5 months OS 28 months
Previous anti-BCMA treatment	Subsequent Bela SA/general population (n°)	Outcomes (vs general population)
CAR-T (real-world) (78)	22/NA	ORR 18%
Any anti-BCMA (real world) (60)	18/94	ORR 29% (vs 43%), PFS 2 (vs 2.8) months, OS 20.4 (vs 17.2) months
Any anti-BCMA (real world) (27)	12/81	ORR 17% (vs 40%)

Bela, belantamab mafodotin; SA, single agent; ORR, overall response rate; DoR, duration of response; PFS, progression free survival; OS, overall survival; NA, not available.

It is important to acknowledge that single agent belantamab mafodotin is associated with lower ORR and duration of response (DoR) compared to bsAbs or CAR T-cell therapies, with a median of 10–11 months in real−world cohorts (60). In contrast, BCMA-targeted CAR T-cell therapies achieve more durable remissions, with PFS exceeding 20, while teclistamab demonstrates a DoR of approximately 24 months in clinical practice (61). This difference affects not only PFS and OS but also the total cost burden for healthcare systems, which must manage more frequent hospital visits and additional drug administrations. Recent evidence suggests that dose delays for bsAbs can be implemented thanks to their durable responses, although official dosing schedules remain unchanged. In line with this, introducing belantamab earlier in the treatment course aims to exploit a wider therapeutic window and reduce dosing frequency, potentially improving both clinical outcomes and economic sustainability.

According to the EMN–EHA recommendations in first relapse, belantamab mafodotin–based combinations retain a defined role in selected clinical scenarios (14). Bela-Vd is recommended in patients with bortezomib-sensitive disease who have previously received lenalidomide and have not developed refractoriness to proteasome inhibition [I, A]. Bela-Pd is recommended in lenalidomide-refractory patients, including those with disease refractory to both lenalidomide and bortezomib or both lenalidomide and daratumumab [I, A]. The recent approval by the FDA of the teclistamab–daratumumab combination for patients RRMM (62) who have received at least one prior line of therapy, including a Pi and an IMiD, introduces an additional competitive option to belantamab-based combination strategies in earlier lines of treatment. With respect to advanced immunotherapies, belantamab-based regimens should be considered preferentially in patients who are not eligible for CAR T-cell therapy due to age, comorbidities, aggressive disease requiring immediate treatment, or limited access to cellular therapies [I, A]. Similarly, in settings where bsAbs (including teclistamab, elranatamab or linvoseltamab) are unavailable, contraindicated, or not immediately accessible, Bela-Vd and Bela-Pd remain effective and rapidly deployable options [I, A] (14).

Although in fit patients who are eligible for CAR T-cell therapy, cellular therapy should be prioritized and not considered a strategy following failure of belantamab mafodotin, high costs and prolonged manufacturing times must also be taken into account, as these factors may delay treatment initiation in patients with rapidly progressive disease (63). Moreover, patients might be ineligible for CAR T-cell therapy because of frailty or comorbidities, such as cardiac disease or neurological disorders. Data from registrational trials suggest that belantamab mafodotin may represent a suitable option for older or unfit patients, given the relatively low incidence of severe infections observed with the single agent and the manageable safety profile, particularly the ocular toxicity, which can be effectively controlled through dose reductions or delays, prophylactic eye drops, and structured ophthalmological monitoring (11, 12). The incidence of severe infections with belantamab-based combinations, especially Bela-Pd, is still not negligible. Importantly, the use of intravenous or subcutaneous immunoglobulin replacement therapy has been shown to reduce both the incidence and severity of infections with other anti-BCMA therapies, including CAR T-cell products and bsAbs, even in earlier lines and in older patient populations (64). Immunoglobulin replacement in patients treated with belantamab-based regimens may similarly contribute to mitigate further infection risk, thereby improving their tolerability and applicability.

Belantamab mafodotin–based strategies have demonstrated to be a convenient treatment option suitable for administration in the outpatient setting, especially for vulnerable patients (65). Unlike other BCMA-targeted therapies, including CAR T-cell products and BsAbs, belantamab-based triplets do not require administration or monitoring in specialized centers, as opportunistic infections were infrequent and neither cytokine release syndrome (CRS) nor immune effector cell–associated neurotoxicity syndrome (ICANS) were reported (7). Nevertheless, the implementation of outpatient step-up dosing protocols for bsAbs, including the use of prophylactic tocilizumab, is expected to become increasingly feasible soon, with acceptable rates of CRS/ICANS and hospitalization without compromising safety (66).

## 3 Other ADCs

ADCs have long been an established and well-recognized therapeutic modality in clinical medicine, both in hematologic malignancies (e.g., polatuzumab in lymphomas (67)) and in solid tumors (e.g., trastuzumab in breast cancer (68)). They differ not only for the target, but also for the payload (alkylating agents, toxins, radiopharmacons etc).

To date, belantamab mafodotin remains the only ADC in multiple myeloma with phase III clinical development and regulatory approval by major agencies. Its clinical trajectory has therefore established proof of principle for the ADC platform, while simultaneously highlighting the challenges faced by other agents in this class. Between anti-BCMA ADCs, alternative payloads were investigated (**Table 3**): MEDI2228 (69) and HDP-101 (70) are linked to tesirine and alpha-amanitin, respectively. MEDI2228 has showed a median ORR of ~ 39%, but G3–4 thrombocytopenia, ocular toxicity and altered liver function was commonly reported; HDP-101, after some steps of dose optimization, demonstrated a median ORR of 36% with no signs of ocular or renal toxicity, G3–4 myelosuppression or liver damage, highlighting the potential of alternative payloads to overcome one of the main limitations associated with first-generation ADCs such as belantamab mafodotin, and supports continued development of next-generation constructs.

**Table 3:** Current status of the main ADCs under development against Multiple Myeloma (MM).

Agent	Target	Mechanism of action/payload	Clinical phase	Findings
MEDI2228 (69)	BCMA	Anti-BCMA antibody conjugated to PBD (tesirine) → internalization, release of PBD causing DNA cross-linking → apoptosis	Phase I, data published 2020–2021; development discontinued due to toxicity	Clinical activity in R/R MM, limited by ocular/systemic toxicity.
HDP-101 (70)	BCMA	Anti-BCMA antibody conjugated to α-amanitin → inhibition of RNA polymerase II, blocking transcription and inducing apoptosis.	Phase I/IIa, ongoing (dose escalation active in 2024–2025); FDA Fast Track designation (2025)	Novel payload; potential activity in high-risk MM (e.g. del17p)
Mokadafusp (75)	CD38	Anti-CD38 antibody fusion protein with attenuated interferon-α (not a classical cytotoxic payload) → immune activation and anti-proliferative signaling	Phase I/II, studies conducted 2019–2024; development partially discontinued	Better described as an immunocytokine rather than a classical ADC
STRO-001 (76)	CD74	Anti-CD74 antibody conjugated to maytansinoid (MMAF) → microtubule inhibition → apoptosis	Phase I, active/recruiting (2023–2025).	Site-specific ADC with manageable toxicity in early trials
FOR46 (76)	CD46	Anti-CD46 antibody conjugated to MMAF → microtubule inhibition → cell death	Phase I, active/recruiting (2023–2025)	Activity correlated with high CD46 expression (e.g. gain 1q MM)
Indatuximab ravtansine (71)	CD138	Anti-CD138 antibody + DM4 (maytansinoid) → microtubule disruption; tested in combination with lenalidomide/dexamethasone	Phase I/II, completed (2015–2018); development discontinued	Limited clinical activity as monotherapy; clinical activity in combination
Lorvotuzumab mertansine (74)	CD56	Anti-CD56 antibody + DM1 (maytansinoid) → microtubule inhibition	Phase I/II, completed (2010–2014)	Limited clinical activity; development not continued

ADC, Antibody Drug Conjugated; CD, Cluster Differentiation.

Other anti-myeloma ADCs have explored alternative targets and payloads (**Table 3**), although none have achieved comparable clinical impact. Indatuximab ravtansine (71) (CD138-directed) represents the most clinically advanced non-antiBCMA ADC. However, its activity as monotherapy was minimal, with very low response rates (ORR 6%) in early-phase studies. While combination trials with lenalidomide- or pomalidomide-based regimens (Rd or Pd) reported higher overall response rates, these outcomes were largely comparable to those historically achieved with the correspondent doublets (72) (especially Rd), suggesting limited additive benefit from the ADC component. Moreover, these studies were characterized by small sample sizes and were conducted prior to the POLLUX (73) era, before the incorporation of daratumumab demonstrated markedly superior efficacy. Importantly, the contemporary treatment landscape further limits the relevance of these findings, as lenalidomide is now commonly used in earlier lines, rendering it less available or ineffective for combination strategies in the relapsed/refractory setting.

**Figure 1:**
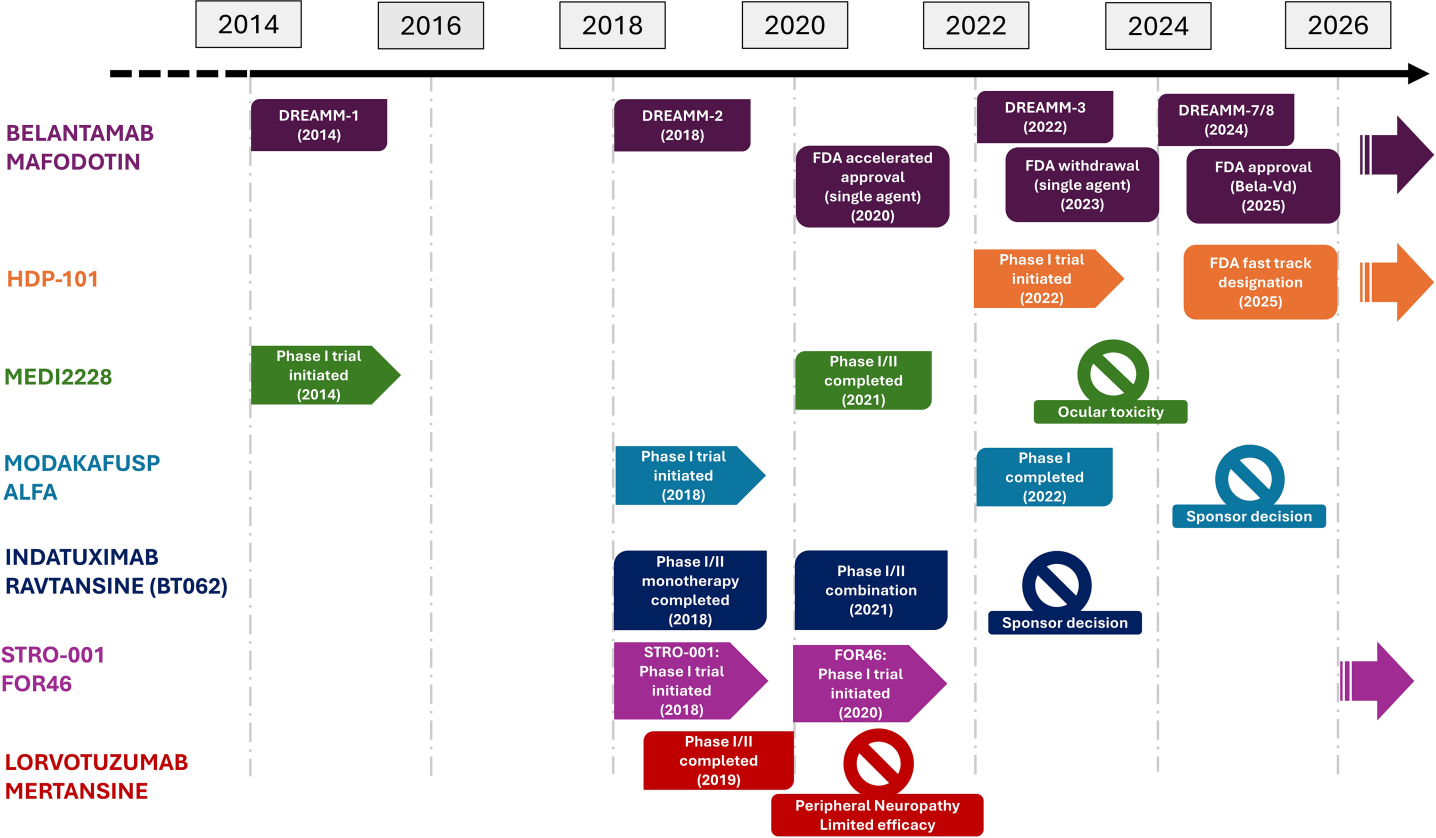
Anti-myeloma ADCs’ development timeline.

Other targets have been tested, for example with Lorvotuzumab mertansine (74) (CD56-directed), Modakafusp (75) (CD38-directed), STRO-001 (CD74-directed), and FOR46 (CD46-directed) (76). Although some of these agents showed preliminary signals of activity, including responses in heavily pretreated or high-risk populations, their development has been hampered by limited durability of response, safety concerns, or immature clinical data, ultimately leading to program discontinuation or early-phase stagnation (**Figure 1**). Nevertheless, some strategies may help overcome the limitations observed with first-generation constructs. In particular, the use of alternative payloads may reduce class-specific toxicities, especially ocular events, as suggested by the HDP-101 experience. In addition, the investigation of non-BCMA targets may have relevant clinical implications, as it could enable the use of ADCs in sequence or in combination with BCMA-directed immunotherapies, including CAR-T cells and bispecific antibodies; however, the mechanisms of resistance associated with these alternative targets remain poorly understood, as does their potential impact on subsequent responses to other therapies.

Finally, it is important to cite the DREAMM-20 trial experience, where belantamab has been tested on RRMM patients without its classical payload, just like a common MoAb (77). The aim was to find a payload-independent anti-tumor efficacy avoiding the mafodotin’s ocular toxicity. Preliminar data show a median ORR of 28% (near to DREAMM-2) with a single patient (6%) who had blurred vision, apparently not therapy-related.

Collectively, these experiences underscore that, outside of belantamab mafodotin, ADC development in multiple myeloma has yet to translate biological rationale into durable clinical benefit, particularly in comparison with rapidly evolving BCMA-directed cellular and bispecific immunotherapies.

## 4 Conclusions

Among ADCs currently under investigation in MM, belantamab mafodotin remains the most extensively studied and clinically implemented BCMA-targeted ADC, demonstrating a favorable balance between efficacy, safety, and outpatient feasibility. Belantamab mafodotin represents an effective and rapidly deployable option for patients with RRMM, particularly for those ineligibles for cellular therapies such as CAR T-cells or bsAbs due to age, comorbidities, rapid disease progression, or limited access to specialized centers. Monitoring BCMA expression and, where feasible, soluble BCMA levels may be recommended to optimize sequencing decisions. With a manageable safety profile and suitability for outpatient administration, Bela-Vd and Bela-Pd remain practical options in selected clinical scenarios, while prioritizing anti-BCMA CAR T-cell therapy when available.

Ongoing studies evaluating belantamab mafodotin in earlier lines of therapy, including the frontline setting, as well as the development of novel ADCs with alternative targets and payloads, may further consolidate the role of this therapeutic class. Ensuring that these advances translate into equitable access to care will be essential to maximize their impact on patient outcomes worldwide.
